# Multidisciplinary intensive lifestyle intervention improves markers of nonalcoholic fatty liver disease (NAFLD) in patients with type 1 diabetes and obesity: a retrospective matched-cohort study

**DOI:** 10.1186/s40842-023-00150-9

**Published:** 2023-04-12

**Authors:** Shaheen Tomah, Tareq Salah, Marwa Al-Badri, Shilton Dhaver, Hannah Gardner, Mhd Wael Tasabehji, Osama Hamdy

**Affiliations:** 1 Joslin Diabetes Center, Boston, MA 02215 USA; 2 Harvard Medical School, Boston, MA 02215 USA

**Keywords:** NAFLD, Type 1 Diabetes, Liver fibrosis, Obesity, Lifestyle intervention, Weight management

## Abstract

**Background:**

The prevalence of non-alcoholic fatty liver disease (NAFLD) is increasing among patients with type 1 diabetes (T1D) paralleling the increasing prevalence of obesity among this population. However, little is known about the impact of intensive lifestyle intervention (ILI) on NAFLD in patients with T1D.

**Methods:**

Using Hepatic Steatosis Index (HSI), a noninvasive surrogate predictor of NAFLD, we retrospectively evaluated 88 adult patients with T1D and obesity after one year of participating in a 12-week ILI program in real-world clinical practice. Using the NAFLD guidelines of the American Association for the Study of Liver Diseases (AASLD), we excluded 11 participants. We matched the remaining ILI cohort (age 43 ± 12 years, females 65%, diabetes duration 22 ± 9 years, A1C 8.2 ± 0.9%, body weight 101 ± 17 kg, BMI 35.3 ± 4.9 kg/m^2^) in 1:1 ratio with a similar cohort of patients with T1D and obesity who received standard diabetes care (SC) at the same practice and during the same period. Matching criteria included: sex, age, BMI, A1C and duration of T1D. HSI [8 + ALT/AST + BMI (+ 2 if female, + 2 if T2D)] was calculated at baseline and after 12 months of intervention.

**Results:**

At baseline, HSI was similar between the two cohorts (46.2 ± 6.1 in the ILI cohort and 44.9 ± 5.7 in the SC cohort). After 12 months, the ILI group lost an average of 5.6 ± 2.7 kg (5.8%, *p* < 0.05) while the SC group maintained their baseline body weight (*p* < 0.001 between groups). HSI decreased significantly from baseline in the ILI group (-2.7 ± 1.1, p = 0.01), but did not change in the SC group (0.6 ± 0.9, p = 0.53, *p* < 0.001 between groups). Percentage of patients with high likelihood of NAFLD diagnosis decreased from 100% at baseline to 88.3% in the ILI group, and was 10.4% less compared to SC (*p* < 0.01). Total daily insulin dose decreased in the ILI cohort compared to the SC cohort (-6.1 ± 4.2 versus 1.34 ± 4.3 units/day, *p* < 0.01).

**Conclusions:**

Twelve weeks of ILI improved HSI and decreased total daily insulin requirements in patients with T1D and obesity at one year. Short-term ILI should be implemented in the management of NAFLD for obese patients with type 1 diabetes.

## Introduction

Non-alcoholic fatty liver disease (NAFLD) is currently considered the most common chronic liver disease [1]. It is strongly associated with obesity, type 2 diabetes (T2D) and metabolic syndrome (MeS). However, NAFLD is also found in non-obese patients without insulin resistance [2]. Its prevalence is increasing among patients with type 1 diabetes (T1D), paralleling the increasing prevalence of obesity among this population [3]. Recent studies reported that up to 50% of patients with T1D are overweight or obese, and between 8–40% meet the MetS criteria [4]. Some studies estimated that the prevalence of NAFLD in patients with T1D is up to 44.4%. However, these studies had small sample sizes, they also showed an association between NAFLD and increased incidence of cardiovascular events, chronic kidney disease, and retinopathy [5–8]. As of now, there are no approved pharmacologic treatments for NAFLD, and weight reduction through dietary and exercise modifications remains the prime intervention tool [9].

Screening for NAFLD in the current clinical practice is frequently conducted using imaging modalities; mainly ultrasonography (US) [10], however the sensitivity of this method decreases when hepatic fat content is < 33% [11]. Other modalities include computed tomography (CT), proton magnetic resonance spectroscopy and magnetic resonance elastography [12]. However, these tools are expensive, and are not cost-effective for screening large populations. Many non-invasive biomarkers have been developed to predict NAFLD, such as the hepatic steatosis index (HSI) which is helpful for early diagnosis in order to provide opportunities for preventing and treating NAFLD and its associated complications [3, 13].

Due to scarce information on the effect of intensive lifestyle intervention (ILI) in obese patients with T1D and NAFLD, we conducted this retrospective study to evaluate the impact of ILI on hepatic steatosis. We selected a cohort of obese patients with T1D who were diagnosed with NAFLD using HSI and underwent an ILI program for 12 weeks, and followed them with a matched control for one year using HSI.

## Methods

The selected cohort for this study include obese patients with T1D who underwent the Weight Achievement and Intensive Treatment (Why WAIT) program at Joslin Diabetes Center in Boston, MA. Why WAIT is a 12-week multidisciplinary ILI program, designed for patients with diabetes and obesity in real-world clinical practice. The main components of the Why WAIT program include the following: 1) intensive medication adjustments; 2) structured modified dietary intervention; 3) individualized exercise plan; 4) cognitive behavioral support; and 5) adult group education. The program showed maintenance of weight reduction for 5 years [14]. A detailed description of the program was described elsewhere [14, 15].

### Study subjects

After approval from the Institutional Review Board of the Joslin Diabetes Center, we retrospectively evaluated all adult patients with T1D and obesity who were enrolled in the Why WAIT program between September 2005 and May 2018. Eighty-eight patients met the criteria for this study. Using the NAFLD guidelines of the American Association for the Study of Liver Diseases (AASLD), we excluded 11 participants mainly due to alcohol consumption of > 21 standard alcoholic drinks per week for men and > 14 for women [16]. The remaining 77 patients were included in this analysis (ILI cohort: mean age 43 ± 12 years, females 65%, diabetes duration 22 ± 12 years, A1C 8.2 ± 1.0%, body weight 102.5 ± 17.3 kg, BMI 35.7 ± 5.0 kg/m^2^). We matched this ILI cohort, in 1:1 ratio, with a similar cohort of patients with T1D who received standard diabetes care (SC) at the same practice and during the same period. Matching criteria included: sex, age, BMI, A1C and duration of T1D (SC cohort: n = 77, mean age 43 ± 12 years, females 65%, diabetes duration 22 ± 9 years, A1C 8.2 ± 0.9%, body weight 100.9 ± 17.2 kg, BMI 35.3 ± 4.9 kg/m^2^). Diagnosis of T1D was confirmed by the presence of auto-antibodies associated with T1D development, history of diabetic ketoacidosis (DKA), very low or undetectable serum C-peptide, and additional relevant clinical criteria. HSI was calculated as [8 + ALT/AST + BMI (+ 2 if female, + 2 if T2D)] at baseline and after 12 months of intervention [17]. Other data collected at baseline and after 12 months for both cohorts included method of insulin delivery (MDI or insulin pump therapy), total insulin dose (units), weight-adjusted insulin dose (units/kg/d), blood pressure, and serum lipid profile.

### Statistical analyses

All tests of group differences were based on the intent-to-treat principle using all available data. Since clinic visits during the follow-up period were not rigorously scheduled every 3 months, an approximation of each visit time to the nearest 3-month timeline was used. There was no evidence that missing data were dependent on the study group. Demographic and baseline characteristics were evaluated using descriptive statistics. Continuous variables are reported as mean ± standard deviation (SD) or standard error of the mean (SEM). Normality of the data was tested using the Kolmogorov-Smirnoff equality-of-distributions test. Categorical variables are presented as percentages. For comparisons between the two study cohorts, independent samples Student's t-test was used for continuous variables. Chi-square or Fisher’s exact tests were used to compare categorical variables. A *p*-value of < 0.05 was considered statistically significant. To determine the possible influence of baseline differences on the study outcomes, statistical analyses were run with and without adjusting for baseline age, BMI, T1D duration, method of insulin delivery, and HSI. We found that adjusting for these variables had no effect on the direction or the significance of study conclusions. Statistical analysis was conducted using SAS version 9.4 for Windows (SAS Institute, Cary, NC, USA 2012).

## Results

Both ILI and SC cohorts were similar at baseline for age, sex, duration of diabetes, A1C, body weight, and BMI (Table 1). HSI at baseline was also comparable between the two cohorts (HSI 46.2 ± 6.1 in the ILI cohort and 44.9 ± 5.7 in the SC cohort). Total daily insulin dose, and insulin requirements per kg of body weight were not significantly different between the two cohorts. The only significant difference between them at baseline was the method of insulin delivery, where the percentage of patients using insulin infusion pumps was significantly higher in the ILI cohort compared to the SC cohort (63.3% versus 46.7%, *p* < 0.05).

**Table 1:** Baseline characteristics of ILI and SC cohorts of patients with type 1 diabetes and obesity

	**ILI** **(*n* = 77)**	**SC** **(*n* = 77)**	***p* value**^**†**^
Age (years)	43 ± 12	43 ± 12	NS
Female n (%)	50 (65)	50 (65)	NS
Diabetes duration (years)	22 ± 12	22 ± 9	NS
Weight (kg)	102.5 ± 17.3	100.9 ± 17.2	NS
BMI (kg/m^2^)	35.7 ± 5.0	35.3 ± 4.9	NS
ALT (U/L)	26.8 ± 20.2	21.6 ± 8.5	< 0.05
AST (U/L)	22.4 ± 9.3	21.7 ± 12.0	NS
HbA1c (%)	8.2 ± 1.0	8.2 ± 0.9	NS
HSI	46.2 ± 6.1	44.9 ± 5.7	NS
HSI > 36 n (%)	77 (100)	75 (97.4)	NS
HSI < 30 n (%)	0 (0)	0 (0)	-
Total insulin dose (units/d)	69.9 ± 27.5	70.8 ± 27.4	NS
Weight-adjusted insulin dose (units/kg/d)	0.7 ± 0.2	0.7 ± 0.2	NS
Use of insulin pump therapy n (%)	49 (63.6)	36 (46.7)	< 0.05
Systolic blood pressure (mmHg)	128 ± 14	126 ± 15	NS
Diastolic blood pressure (mmHg)	77 ± 8	77 ± 9	NS
Total Cholesterol (mg/dL)	169 ± 33	176 ± 26	NS
LDL-Cholesterol (mg/dL)	97 ± 32	97 ± 27	NS
HDL-Cholesterol (mg/dL)	55 ± 16	58 ± 17	NS
Triglycerides (mg/dL)	114 ± 88	121 ± 73	NS

Data are mean ± SD

*ILI* Intensive lifestyle intervention, *SC* Standard diabetes care, *HSI* Hepatic steatosis index, *NAFLD* Nonalcoholic fatty liver disease, *ALT* Alanine aminotransferase, *AST* Aspartate aminotransferase

^†^Student’s t-test or Pearson’s chi squared between groups

**Fig. 1:**
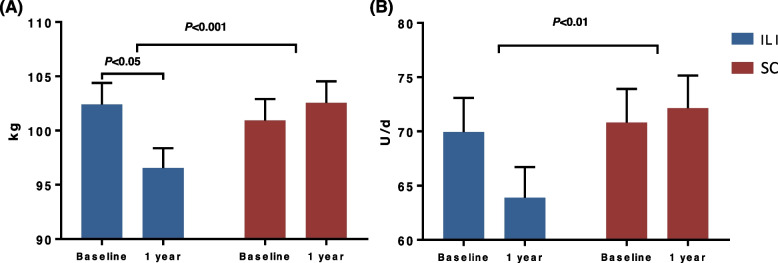
Changes in (**A**) body weight and (**B**) total daily insulin dose after 1 year. Data are mean±SD. ILI, Intensive lifestyle intervention; SC, Standard diabetes care. n=77 in each cohort

**Fig. 2:**
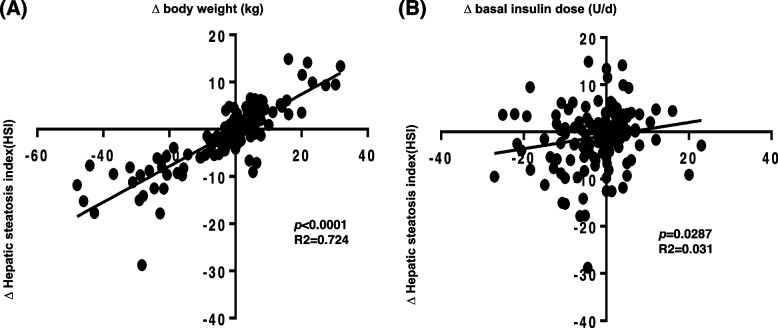
Change in HSI, body weight, and daily basal insulin dose after 1 year. Legend. **A** Association between change in HSI and change in body weight after 1 year in response to ILI and SC in the total cohort. **B** Association between change in HSI and daily basal insulin dose after 1 year in response to ILI and SC in the total cohort. HSI, Hepatic steatosis index; ILI, Intensive lifestyle intervention; SC, Standard diabetes care. N=154

**Fig. 3:**
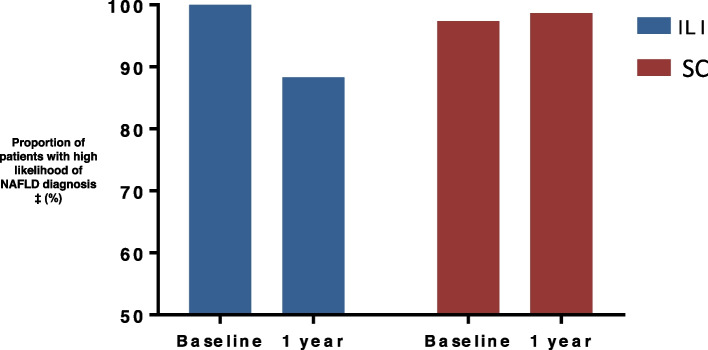
Likelihood of NAFLD diagnosis after 1 year ILI and SC. Data are %. n=77 in each cohort. NAFLD, nonalcoholic fatty liver disease. ‡ High likelihood of NAFLD diagnosis defined as having HSI>36. HSI>36 can detect NAFLD with a specificity of 92.4% and a positive likelihood ratio of 6.069

After 12 months, the ILI group lost an average of 5.6 ± 2.7 kg (5.8%, *p* < 0.05) while the SC group maintained their baseline body weight (*p* < 0.001 between groups) (Fig. 1). HSI decreased significantly from baseline in the ILI group (-2.7 ± 1.1, p = 0.01), but did not change in the SC group (0.6 ± 0.9, p = 0.53, *p* < 0.001 between groups) (Table 2). Changes in HSI correlated significantly with the change in body weight and the daily basal insulin dose (Fig. 2). Moreover, the percentage of patients with high likelihood of NAFLD diagnosis decreased from 100% at baseline to 88.3% at 1 year in the ILI group and was 10.4% less compared to SC (98.7%) (*p* < 0.01) (Fig. 3). Changes in body weight, BMI, and HSI remained significant in the ILI cohort after adjusting for age, sex, T1D duration, and method of insulin delivery.

**Table 2:** Changes after one year of ILI or SC in patients with T1D and obesity

	**ILI** **(*n* = 77)**	**SC** **(*n* = 77)**	***p* value**^**†**^
Weight (kg)	-5.6 ± 2.7*	1.6 ± 2.8	< 0.001
BMI (kg/m^2^)	-1.8 ± 0.9*	0.6 ± 0.8	< 0.01
ALT (U/L)	-4.0 ± 2.5	1.9 ± 1.3	< 0.05
AST (U/L)	-0.6 ± 1.1	0.2 ± 1.6	NS
HbA1c (%)	-0.33 ± 0.15*	-0.07 ± 0.16	NS
Total daily insulin dose (units/d)	-6.1 ± 4.2	1.3 ± 4.3	< 0.01
Weight-adjusted insulin dose (units/kg/day)	-0.014 ± 0.020	0.004 ± 0.013	NS
Systolic blood pressure (mmHg)	-3.6 ± 2.1	0.4 ± 2.5	NS
Diastolic blood pressure (mmHg)	-1.9 ± 1.3	-0.1 ± 1.4	NS
Total cholesterol (mg/dL)	0.1 ± 6.3	0.9 ± 5.2	NS
LDL-cholesterol (mg/dL)	-4.7 ± 5.4	0.2 ± 3.4	NS
HDL-cholesterol (mg/dL)	1.1 ± 2.9	-0.9 ± 1.0	NS
Triglycerides (mg/dL)	-7.7 ± 13	-0.4 ± 9.0	NS
HSI	-2.7 ± 1.1*	0.6 ± 0.9	< 0.001

Date are mean ± SEM

*ILI* Intensive lifestyle intervention, *SC* Standard diabetes care, *HSI* Hepatic steatosis index, *ALT* Alanine aminotransferase, *AST* Aspartate aminotransferase

^*^p < 0.05 compared to baseline

At 12 months, ALT improved in the ILI cohort compared to baseline (ALT change of -4.0 ± 2.5 U/L) with no change in the SC cohort (*p* < 0.05 between groups). However, the difference in AST between the two cohorts was not significant.

Total daily insulin dose decreased significantly from baseline in the ILI cohort compared to the SC cohort (-6.1 ± 4.2 versus 1.3 ± 4.3 units/day, *p* < 0.01). Difference in daily insulin dose remained significant after adjusting for age, sex, BMI, T1D duration and method of insulin delivery. However, weight-adjusted insulin requirements did not change in either of the two cohorts at 12 months.

A1C, blood pressure, and lipid profile showed no significant changes in either of the two cohorts at 12 months.

## Discussion

Prior research showed that NAFLD develops as a possible consequence of hyperglycemia, insulin resistance, and obesity [18], however little research has been done to investigate strategies to mitigate NAFLD in patients with T1D and obesity.

This study showed that ILI intervention in real-world clinical practice for 12 weeks, is associated with an improvement in HSI in patients with T1D and obesity at one year in comparison to standard diabetes care that typically focuses on glycemic control. By the end of 12 months, patients who underwent ILI had a significantly lower percentage of NAFLD as diagnosed by HSI calculation, indicating possible resolution of the condition in some patients after such intervention. The study also showed improvement in ALT which is another component of HSI, indicating an improvement in liver fat. Moreover, there was a reduction in total insulin dose, which might indicate improvement in insulin sensitivity after weight reduction. Our research team previously demonstrated that ~ 7% weight reduction in patients with insulin resistance improves insulin sensitivity by ~ 57% [19]. The amount of weight loss that resulted in such improvement in this study is an average of 5.6 ± 2.7 kg (5.8%), which is significantly better than weight reduction by standard diabetes care for one year, which was 1.6 ± 2.8 kg (1.6%). Our observed results draw attention to the importance of a short period of ILI for 12 weeks as a possible modifier of NAFLD at a longer term of one year.

Other research work has demonstrated that NAFLD is potentially reversible by pharmacological intervention [20]. However, to our knowledge, this is the first study to explore the longevity of NAFLD improvement or resolution at one year after a short period of ILI. Since prior research only demonstrated the reversibility of liver fibrosis but not liver cirrhosis [21], detecting NAFLD early enough by screening susceptible patients in real-world clinical practice using simple measurements may lead to saving many cases from progressing to liver cirrhosis through implementing an effective short-term ILI.

The study has many limitations and few strengths. Perhaps the most prominent limitation is our way of diagnosing NAFLD. Although HSI is found to be reliable in determining NAFLD [22], NAFLD can only be truly diagnosed and confirmed by liver biopsy [23]. Since this study was retrospective, it was not possible for us to obtain liver biopsies for either of the two cohorts. Consequently, we cannot be certain that all included patients had NAFLD. The prevalence of NAFLD in patients with T1D is around 20–30% and might be higher in an obese population [24]. Another limitation is the fact that the BMI component of HSI is overpowered compared to that of the liver enzymes. Moreover, these indices are not validated in reflecting prospective longitudinal changes in NAFLD, fat content, and fibrosis [25–28]. However, in real-world clinical practice, a simple screening method may help in detecting more patients who may benefit from early intervention in comparison to the more costly methods. Another limitation is that patients in the ILI cohort voluntarily decided to seek a program to manage their body weight and considered more motivated for change. The retrospective nature of the study is another drawback, since included patients might have other health complications that could have impacted our findings. This warrants another well-designed prospective-controlled study to confirm our observations. Another limitation, was that the two cohorts were confined to patients with T1D and obesity, therefore our findings are not applicable to lean individuals with T1D, a few of whom may also have NAFLD. Lastly, it is hard to rule out the effects of other confounders that may affect the results, whether at baseline or at 12 months. These confounders may include: the use of insulin pump versus injections, which is higher in the ILI cohort, the use of other hypoglycemic medications, and any other medications that may affect weight or liver fat content. These drawbacks may limit the generalizability of our results to all patients with T1D and obesity. However, despite these limitations, we were able to uncover that short-term weight loss through ILI may be an effective tool for improving NAFLD parameters in patients with T1D and obesity. On the positive side, this study was conducted among patients in real-world clinical practice, which may indicate its potential value for direct application in diabetes practice, especially with its simpler screening method and available ILI programs in many clinical centers.

In conclusion, short-term ILI leads to a consequential improvement in NAFLD indices in patients with T1D and obesity and is associated with a reduction in insulin requirements. Even though HSI is not widely used for monitoring the progression of steatosis, it is a simple method for NAFLD screening and initiating ILI that results in weight reduction and potential improvement of hepatic steatosis. Therefore, we recommend a short-term ILI for obese patients with T1D and NAFLD, while calling for other prospective-controlled studies to be conducted in order to confirm our notion.

## Data Availability

The data contained in this manuscript are held at the Joslin Diabetes Center Clinical Research Center. The datasets used and/or analyzed during the current study are available from the corresponding author upon reasonable request.

## Abbreviations

NAFLD: Nonalcoholic fatty liver disease
T1D: Type 1 diabetes
T2D: Type 2 diabetes
HSI: Hepatic steatosis index
ILI: Intensive lifestyle intervention
AASLD: American Association for the Study of Liver Diseases
SC: Standard diabetes care
MetS: Metabolic syndrome
NASH: Nonalcoholic steatohepatitis
US: Ultrasound
CT: Computed tomography
SMBG: Self-monitoring of blood glucose
EP: Exercise psychologists
BMI: Body-mass index
DKA: Diabetic ketoacidosis
ALT: Alanine aminotransferase
AST: Aspartate aminotransferase
MDI: Multiple daily injections
